# Targeted solutions to increase dolutegravir coverage, viral load testing coverage, and viral suppression among children living with HIV in Togo: An analysis of routine facility data

**DOI:** 10.1371/journal.pone.0296293

**Published:** 2023-12-21

**Authors:** Caterina Casalini, Yema D’Almeida, Moussa Ariziki Nassam, Essopha Kokoloko, Souley Wade, Jean Paul Tchupo, Messan Damarly, Justin Mandala, Michele Lanham, Natasha Mack, Chris Akolo, Vincent Polakinam Pitche, Hugues Guidigbi, Claver Anoumou Dagnra

**Affiliations:** 1 HIV Programs, FHI 360, Hilversum, Netherlands; 2 WAMERO, FHI 360, Lomé, Togo; 3 HIV Programs, FHI 360, Durham, NC, United States of America; 4 National AIDS Council, Lomé, Togo; 5 USAID/West Africa, Accra, Greater Accra Region, Ghana; 6 National AIDS Program, Lomé, Togo; United States Agency for International Development (USAID), NIGERIA

## Abstract

**Background:**

According to UNAIDS, Togo halved AIDS-related deaths among children ages 0–14 from 2010 to 2020. However, available data show low dolutegravir (DTG)-containing antiretroviral therapy (ART) coverage and low viral load suppression (VLS) among children living with HIV (CLHIV). We analyzed routine facility data before and after implementation of root-cause-based solutions for improving DTG coverage, viral load (VL) testing coverage, and VLS among CLHIV.

**Description:**

We analyzed routine data for CLHIV ≤14 years from October 2019 through September 2022. We assessed proportion of CLHIV on ART receiving DTG, VL testing coverage (CLHIV on ART with documented VL test result), and VLS (CLHIV with documented VL test result of <1,000 copies among those with test result). From October 2019 to September 2020, 52% were on a DTG-containing regimen, 48% had documented VL test results, and 64% had VLS. Site-level teams conducted a root-cause analysis and designed corresponding solutions implemented beginning October 2020: line listing and contacting eligible CLHIV to start/transition to DTG-containing regimen and collect VL samples; ART adherence support; monthly DTG stock monitoring; tracking pending VL test results through laboratory focal persons; documenting VL test results; and informing caregivers within one week if CLHIV not virally suppressed. Granular data were used to prioritize technical assistance to sites with lowest DTG coverage, VL testing coverage, and VLS.

**Results:**

From baseline (October 2019–September 2020) to endline (October 2021–September 2022), increases were observed for DTG coverage (52% to 71%), VL testing coverage (48% to 90%), and VLS (64% to 82%). Age-disaggregated data showed positive trends.

**Conclusions:**

Root-cause-based solutions and granular data use increased DTG coverage, resulting in increased VL testing and VLS among CLHIV. These interventions should be scaled and become the national standard of care.

## Introduction

The Western and Central Africa regions continue to make progress toward the UNAIDS 95–95–95 targets. As of 2021, 80% of people living with HIV (PLHIV) knew their HIV status, 98% of people who knew their HIV-positive status were accessing treatment, and 88% of people on treatment had suppressed viral load (VL). However, children living with HIV (CLHIV) ages 0–14 years are being left behind: only 35% had access to antiretroviral therapy (ART) in 2021, and only 27% were virally suppressed. In Togo, the Joint United Nations Programme on HIV/AIDS (UNAIDS) estimated 8,700 CLHIV in 2021, of whom 49% knew their status, 49% were on treatment, and 35% were virally suppressed, and it estimated 1,000 new HIV infections among children ages 0–14 years. Children also continue to experience a disproportionate share of AIDS-related deaths, accounting for 4% of PLHIV globally but 15% of AIDS-related deaths in 2021. In Togo, children represented 8% of PLHIV, one-third of new infections, and about one-quarter of AIDS-related deaths [1].

The lack of safe, effective, and simplified ART regimens with good tolerability has been a barrier to achieving treatment success among CLHIV, but findings from the ODYSSEY (PENTA 20) [2] and IMPAACT P1093 [3] trials showed superiority, effectiveness, safety, and high tolerability of the integrase inhibitor dolutegravir (DTG) among CLHIV.

These results paved the way for the World Health Organization (WHO)’s 2018 recommendation to use DTG as first-line ART for all PLHIV, including children over the age of 4 weeks and weighing ≥3 kg [4], providing an opportunity to stop using regimens with high toxicity, low genetic barriers to resistance, and cold chain requirements, and antiretrovirals (ARVs) in the form of unpalatable syrups or bitter, hard-to-swallow solid pediatric formulations taken multiple times per day [5], and to move toward DTG-containing regimens. Similarly, the U.S. President’s Emergency Plan for AIDS Relief (PEPFAR) Country Operational Plan (COP) 22 recommends the scale-up of a fixed-dose combination of tenofovir, lamivudine, and DTG (TLD) for all PLHIV ≥3 kg and ≥4 weeks old. As a result, the integrase inhibitor DTG has been introduced as an alternative to efavirenz (EFV) in first-line treatment, and PEPFAR has ceased procurement of EFV-based treatment [6].

The DTG 50 mg and the new DTG 10 mg dispersible formulations, in combination with other dispersible ARVs, provide treatment that is easier to administer, tastes good, has reduced side effects, and can improve viral suppression. Unfortunately, the COVID-19 pandemic caused major delays in the transition to DTG-containing ART, as well as in VL monitoring, which is encouraged; however, VL testing and documentation of suppressed VL should not be a requirement for transitioning to TLD. VL testing should instead be prioritized following the change in regimen for clients who did not have a documented VL test result or who were not virally suppressed prior to the switch to DTG.

The Ending AIDS in West Africa (#EAWA) project in Togo supports HIV care and treatment services for adults and CLHIV in 13 districts and 19 public health facilities. Following the Ministry of Health’s 2019 recommendations to offer DTG-containing regimens as the first-line treatment for CLHIV, #EAWA identified and rolled out a package of interventions to scale up DTG coverage among CLHIV in all supported health facilities. Here we report the results of our analysis of program data comparing the proportion of CLHIV on a DTG regimen before and after the interventions, as well as other key outcomes.

## Materials and methods

### Setting

Togo has 697 ART clinics, of which 90 are private, 49 are led by nongovernmental organizations, and 558 are public. During the period of analysis (July 2020–September 2022), the #EAWA project supported HIV care and treatment services in 19 of these public health facilities across 13 districts. They were distributed by region as follows: eight of the 30 public facilities in Gran Lome’, four of Maritime’s 136 public facilities, five of the 161 public facilities in Plateau, and two in the Central region out of a total of 86 public facilities. The Kara and Savanes regions were not represented in our study. All 19 facilities whose program data were included in the analysis were located in urban areas.

### Analysis objectives and design

The primary aim of this descriptive analysis was to compare the proportion of CLHIV on a DTG-containing regimen before and after receiving a package of interventions. Secondary outcomes included pre- and post-comparison of VL testing coverage, viral load suppression (VLS), multimonth dispensing (MMD), interruption in treatment, and death among these children.

We analyzed aggregated program data from #EAWA-supported facilities for CLHIV ages 14 years and younger, comparing baseline data (July–September 2019) to endline data (July–September 2022). We did not conduct a statistical analysis to test the causal relationship between the interventions and DTG coverage because we used aggregated data and could not account for confounding factors that could affect the outcomes. Therefore, we used descriptive statistics and graphical methods to explore the data and generate hypotheses rather than inferential statistics to test hypotheses.

We also analyzed the demographic, clinical, and immunological characteristics of the CLHIV. Since we used aggregated data, information about distance from client residence to facilities was not available, nor was information about the urban versus rural distribution of client residence.

### Data collection and analysis

At the site level, data were collected and analyzed on a weekly basis for the purposes of guiding the surge teams on progress and informing decision-making to reach the benchmarks. Once a month, the project coordinator joined the site-level surge team meeting to review aggregated monthly data, discuss the findings and actions from the previous four weeks, and provide technical assistance.

We accessed the routinely collected, aggregated data for this analysis on January 2, 2023. For the purposes of the analysis, data were organized into baseline (July–September 2020) and endline (July–September 2022) periods and by age band (under 5 years, 5–9 years, and 10–14 years).

We used descriptive statistics to summarize the characteristics of the CLHIV, including the number of clients who received CD4 testing. Our calculation of the number of CLHIV on optimized regimens was based on the 2019 national recommendations for optimized regimens, which included the use of DTG 10 mg for children >4 weeks to <6 years and DTG 50 mg in children ≥6 years and weighing ≥20 kg.

We measured the proportion of CLHIV on ART who received a DTG-containing regimen among those currently on ART (defined as having received at least one ART service at the measurement point). We calculated MMD as CLHIV on MMD among those currently on ART, and VL coverage as CLHIV with a documented VL test result within the past 12 months among those on ART for at least six months. VLS was defined as the number of CLHIV having <1,000 copies/mL among those with a documented VL test result within the past 12 months, as per national and WHO guidelines. We also calculated the number of CLHIV who interrupted treatment (defined as having missed the last appointment by ≥28 days) and the number of deaths among those currently on treatment.

As per standard practice, program data were collected using standardized paper-based forms and then entered into the electronic medical record (EMR) system by data entry clerks. De-identified data were then extracted from the database. The extracted data were aggregated and included age, sex, and clinical information such as ART regimen, MMD, most recent VL test result, and status of the client (whether currently on ART, with treatment interruption, or deceased). The analysis also included information from the national supply management system about the monthly stock of EFV and DTG.

### Data quality

All data were regularly validated through the project’s established processes for data quality assurance using data triangulation between the paper tools and EMRs. The data entry clerks reviewed the data on the paper-based forms for completeness and consistency before entering them into the database. Built-in validation rules within the database mitigated data entry and transcription errors. At the end of each day, a gap analyzer was run on the database to identify data errors, which were then checked against the source documents and cleaned. Any gaps and outliers identified were reconciled. Data were summarized and reported daily and monthly.

### Ethical considerations

All patients and their guardians were educated on and informed about DTG before it was offered. All clients provided with the drug gave their verbal informed consent. Those who did not agree to use DTG as prescribed were not eligible for and did not initiate the DTG-containing regimen.

All data included in this paper were extracted from the routine project performance reports, which are publicly available. Our analysis included only aggregated program data used for routine program monitoring and improvement. The authors did not have access to individual-level data or personally identifiable information for the individuals whose data were included in the analysis; all data were fully anonymized prior to researcher access. The request to conduct secondary analysis of program data that did not contain any personally identifiable information was reviewed by the institutional review board of FHI 360 and classified as non-human-subjects research.

### #EAWA interventions

#EAWA followed the national guidelines for the provision of ART to CLHIV. The national guidelines from October 2019 for people living with HIV in Togo recommend abacavir (ABC), lamivudine (3TC), and DTG 10 mg or 25 mg for children >4 weeks to 5 years and DTG 50 mg to children ≥6 years and weighing ≥20 kg [7]. However, although DTG 50 mg became available in November 2019, DTG 10 mg only became available in March 2022, toward the end of the analysis, and DTG 25 mg was not available for the entire period of analysis.

Health care providers at all #EAWA sites (including clinicians, nurses, and other providers who prescribe ARVs) were trained on the new regimen between July 2019 and September 2019. Beginning in January 2020, children newly identified as HIV positive were started on a DTG- containing regimen, and those already on treatment (abacavir [ABC], zidovudine/3TC/lopinavir/ritonavir, or efavirenz) were transitioned to a DTG-containing regimen, regardless of VL status. The CLHIV who were virally suppressed prior transitioning to a DTG-containing regimen repeated the VL test at 12 months after the previous test. Those who were not virally suppressed prior to the transition repeated the VL test at three months from the previous test. This approach aligned to the WHO recommendation that VL testing not be required as a precondition to transition to DTG-based regimens [8], although routine VL monitoring was recommended to deliver appropriate care to CLHIV.

#### Standard #EAWA intervention (baseline through October 2020)

As per the standard of care at baseline and before October 2020, when a package of interventions to scale up DTG coverage was rolled out, the CLHIV at the #EAWA-supported facilities were initiated on ART at the health facility, as the national guidelines did not permit community ART initiation. After the initial month of treatment, they were given three-month refills at the health facility or in the community. Per the national guidelines, only virally suppressed CLHIV were eligible for and offered three- to five-month multimonth dispensing (MMD) of ART.

The CLHIV and caregivers were linked to a mediator, who was a community worker trained in pediatric HIV prevention, care, and treatment and pediatric ART case management, and experienced in providing ART counseling to CLHIV and their caregivers. Each site had one or two mediators according to site client volume and one psychologist, who together were responsible for case management of all ART clients (adults, adolescents, and children under 15).

The mediator reminded the CLHIV/caregiver about and offered navigation to any HIV-related appointment (e.g., for ART refill, VL testing, and any other diagnostic or relevant clinical assessment) through in-person and virtual contact. At the time of the ART refill, the mediator provided in-person ART adherence support and counseling using standardized national tools specific to CLHIV. CLHIV who reported barriers to treatment that could not be addressed by the mediator, as well as CLHIV with unsuppressed VL, were linked to the psychologist. CLHIV who reported side effects and any other clinical issues were linked to the ART health care workers.

The psychologist received ART clients referred by the mediator and provided ART adherence counseling specific for CLHIV, using standardized national tools. CLHIV who were not virally suppressed received monthly therapeutic educational sessions with the psychologist for three months.

Both the psychologist and mediator were based at the ART clinic and did not offer any community-based services. VL sample collection was offered at both the facility and community levels by a laboratory technician and through phlebotomy.

#### #EAWA surge solutions for DTG coverage and VL monitoring scale-up (October 2020–September 2022)

From July through September 2020, only 52% of the CLHIV then on treatment were on a DTG-containing regimen, while 48% of those on treatment had a documented VL test result, of whom 64% had attained VLS. Given these rates, in October 2020, after the health care providers were trained on the optimized regimens and DTG availability was ascertained, site-level surge teams from the health facilities were established. Comprised of ART health care workers (nurses and clinicians), mediator(s), a psychologist, and data managers, these surge teams were tasked with conducting a root-cause analysis of barriers and identifying solutions for scale-up of DTG and VL testing coverage among CLHIV.

The surge teams identified the key barriers to rapid scale-up of the DTG-containing regimen as a lack of the following elements: a dedicated site-level team monitoring DTG and VL testing coverage among CLHIV; tracking of DTG site-level stock based on the expected volume of CLHIV to be initiated and transitioned to the DTG-containing regimen; a systematic approach to line listing clients and offering MMD to eligible CLHIV; line listing CLHIV eligible for a DTG-containing regimen and VL testing; systematic approaches to starting and transitioning CLHIV to the DTG-containing regimen, collecting VL samples, monitoring the return of VL test results, informing clients of results, and assessing barriers to ART adherence; provision of intensified, more frequent ART adherence support to CLHIV and caregivers who reported barriers to treatment; and data use for decision-making. Solutions were then designed to address the specific root causes.

From October 2020 through September 2022, the site-level surge teams supported implementation and monitoring of the agreed-upon solutions to scale up DTG, MMD, and VL monitoring among CLHIV to reach the PEPFAR benchmarks of 95% DTG coverage, 80% MMD coverage, 95% VL testing coverage, and 95% VL suppression among CLHIV. The following solutions were implemented:

- The mediator conducted monthly site-level ART stock monitoring and compared it to the estimated volume of CLHIV to be initiated on or transitioned to a DTG-containing regimen, and those eligible for MMD. If any shortage was anticipated, the mediator alerted the pharmacist of the health facility, who would place a new order through the national supply chain management system, and the project coordinator, who coordinated with the site-level pharmacist to troubleshoot imminent shortages and stock-outs.
- On a monthly basis, the data manager provided the mediator with the line list of CLHIV with an appointment at the ART clinic and those eligible for VL testing and MMD.
- The mediator assisted the ART health care providers to ensure that all CLHIV were initiated on or transitioned to a DTG-containing regimen during the follow-up appointment. Initiation on or transition to the DTG-containing regimen was done by the ART clinician or ART nurse at the ART clinic only, as national guidelines did not permit this at the community level. Virally suppressed CLHIV were given MMD, and their ARV dosing was assessed using a weight-band tool.
- At enrollment into ART services and at every ART refill, the mediator systematically assessed the barriers to ART adherence with CLHIV and caregivers. Those who reported barriers were offered weekly in-person, barrier-specific ART adherence support by the mediator. This targeted strategy of motivational counseling was designed to help CLHIV/caregivers identify their individual barriers to adherence and develop strategies to improve adherence to achieve viral suppression. The counseling meetings were held at the facility or community level (often the CLHIV/caregiver’s residence) according to CLHIV/caregiver preference.
- During the counseling, the mediator discussed the side effects of ARVs and structural barriers such as privacy, stigma and discrimination, fear of disclosure, school attendance, and travel to pick up ART refills, among other themes.
- The frequency of the support decreased from weekly to monthly upon improvement in adherence and compliance with the ARV refill schedule. This was assessed based on self-reported adherence by the CLHIV/caregiver and the pharmacy refill record; a specific adherence metric was not used or documented.
- The mediator followed up with CLHIV/caregivers who missed their ART appointment first via phone call and, if not reached, through home visits.
- All CLHIV/caregivers who missed an appointment and returned to care, as well as those not virally suppressed, were invited to participate in individual or group ART counseling led by the mediator and focused on identifying and addressing barriers and improving adherence to treatment.
- The mediator assisted the laboratory technician to ensure the collection of VL samples for all eligible CLHIV during the follow-up appointment at the facility, or in the community if the CLHIV/caregiver could not come to the ART clinic. If the VL sample was collected at the health facility, this was timed to coincide with the ART refill to avoid the need for the CLHIV/caregiver to return to the facility multiple times. The VL samples were transported by the laboratory technician to a public laboratory on a weekly basis.
- The laboratory technician retrieved the VL test results upon delivery of new samples to the laboratory and returned them to the ART clinic. On a weekly basis, the ART provider contacted the public laboratory focal person to track pending test results that had not been returned after 14 days. The VL test results were entered into the EMR by the data entry clerk, as soon the test result was received at the ART clinic.
- The mediator contacted the caregiver via phone within one week of receipt of the test result at the ART clinic if the CLHIV was not virally suppressed, while for CLHIV who were virally suppressed, the test result was shared at the time of ART refill.
- The site-level surge team met weekly to review and discuss data reports on ART stock, DTG, MMD, and VL coverage, and VLS, and to discuss case-by-case solutions for CLHIV who were reporting barriers to ART adherence.
- The project coordinator participated in the site-level surge team meeting on a monthly basis to provide technical assistance on data review, interpretation, and use for programming improvement; assist with ensuring data documentation and high-quality data; and troubleshoot DTG supply issues. Data were used to prioritize the project coordinator’s technical assistance to focus on ART clinics with the highest volume of CLHIV currently on treatment and the lowest DTG and VL testing coverage, using the 80% and 95% benchmarks, respectively, as the reference thresholds.

Table 1 describes the services provided at baseline (September 2020) and the solutions being implemented at endline (September 2022).

**Table 1:** ART services provided to CLHIV/caregivers at baseline and endline.

	Service	Baseline	Endline
		Sept 2020	Sept 2022
1	ART initiation provided at health facility	X	X
2	Three ART refills of one-month supply provided at the health facility or in the community	X	X
3	3–5 MMD offered to virally suppressed CLHIV	X	X
4	Weight-band tool used for ARV dosing		X
5	CLHIV and caregivers linked to a mediator	X	X
6	Mediator reminded the CLHIV/caregiver about appointments through in-person and virtual contact	X	X
7	Mediator offered in-person ART adherence counseling	X	X
	• At the facility	X	X
	• In the community		X
8	Mediator assessed and discussed in person the specific barriers to ART adherence at enrollment and at every ART refill in the facility and community		X
9	Mediator linked clients with barriers to treatment and those with unsuppressed VL to the psychologist	X	X
10	Psychologist offered in-person enhanced ART adherence counseling and psychological support at the health facility	X	X
11	Mediator changed frequency of support depending on adherence, e.g., decreased or increased frequency upon adherence improvement or worsening, respectively		X
12	Mediator linked clients with side effects and any other clinical issues to the ART health care workers	X	X
13	ART health care workers managed the side effects and any other clinical issues at the health facility	X	X
14	Laboratory technician collected the VL sample at the facility and in the community through full-blood sampling	X	X
15	Mediator assisted the laboratory technician to ensure VL samples were collected from all eligible CLHIV		X
16	ART provider contacted the laboratory focal person to track pending test results		X
17	Mediators contacted the caregiver when the test result was received at the ART clinic if the CLHIV was not virally suppressed		X
18	Mediator conducted monthly site-level DTG stock monitoring		X
19	Project coordinator supported the site-level pharmacists to troubleshoot imminent DTG shortages and stock-outs		X
20	Data manager generated weekly line lists of CLHIV with an appointment at the ART clinic, those who missed an appointment and interrupted treatment, those eligible for VL testing, those virally unsuppressed, and those eligible for MMD		X
	Mediator assisted the ART health care providers to initiate/transition CLHIV to a DTG- containing regimen during the facility-based appointment		X
	DTG 50 mg		X
	DTG 10 mg		X
21	Mediator followed up with the CLHIV/caregiver who did not attend the ART appointment or interrupted treatment via phone calls and home visits		X
22	CLHIV/caregivers who missed an appointment and returned to care and those who were not virally suppressed were invited to participate in individual or group ART counseling led by the mediator		X
23	Site-level surge team met to review and discuss data reports		X
24	Project coordinator participated in the site-level surge team meeting, provided technical assistance to regularly review, interpret, and use data to improve programming, and assisted with ensuring data documentation and data quality		X

## Results

For the period of analysis, the #EAWA project reported 1,723 CLHIV ages 0–14 on treatment at baseline (July–September 2020) and 2,397 at endline (July–September 2022). Their demographic, clinical, and immunological characteristics are provided in Table 2. The sex and age distribution, whereby females represented 52% of the total population and 78% were ages 5–14 years, remained the same from baseline to endline. At baseline, 58% of the CLHIV were WHO stage II and II compared to 75% at endline. WHO staging information was missing for a third of the CLHIV (higher among those under age 5) at baseline, decreasing to 15% at endline. At baseline, a large majority (79%) of the CLHIV did not have a documented CD4 test result, and a similar proportion (82%) was reported at endline; among those with a documented CD4 test result, the majority had <200 cells/mm3 across all age groups.

**Table 2:** Demographic, clinical, and immunological characteristics of CLHIV ages 0–14 years currently on treatment, at baseline and endline.

	Jul–Sept 2020 (baseline)		Jul–Sept 2022 (endline)	
Characteristics	Number	%	Number	%
**Sex at birth**	**1,723**	**100%**	**2,397**	**100%**
Male	823	48%	1,142	48%
Female	900	52%	1,255	52%
**Age band**	**1,723**	**100%**	**2,397**	**100%**
Under 5 years	406	23%	510	21%
5–9 years	600	35%	841	35%
10–14 years	717	42%	1,046	44%
**WHO stage**	**1,723**	**100%**	**2,397**	**100%**
WHO stages I and II	1,011	58%	1,776	75%
WHO stages III and IV	203	12%	251	10%
Missing data	509	30%	370	15%
**Under 5 years**	406	23%	510	21%
WHO stages I and II	178	44%	347	68%
WHO stages III and IV	22	5%	33	6%
Missing data	206	51%	130	26%
**5–9 years**	600	35%	841	35%
WHO stages I and II	354	59%	622	74%
WHO stages III and IV	74	12%	75	9%
Missing data	172	29%	144	17%
**10–14 years**	717	42%	1,046	44%
WHO stages I and II	479	67%	807	77%
WHO stages III and IV	107	15%	143	14%
Missing data	131	18%	96	9%
**CD4 count**	**1,723**	**100%**	**2,397**	**100%**
<200 cells/mm3	352	20%	421	17%
≥200 cells/mm3	12	1%	25	1%
Missing data	1,359	79%	1,958	82%
**Under 5 years**	406	23%	510	21%
<200 cells/mm3	29	7%	34	6%
≥200 cells/mm3	3	1%	3	1%
Missing data	374	92%	473	93%
**5–9 years**	600	35%	841	35%
<200 cells/mm3	121	20%	128	15%
≥200 cells/mm3	5	1%	8	1%
Missing data	474	79%	705	84%
**10–14 years**	717	42%	1,046	44%
<200 cells/mm3	202	28%	259	25%
≥200 cells/mm3	4	1%	7	1%
Missing data	511	71%	780	75%

**Fig 1:**
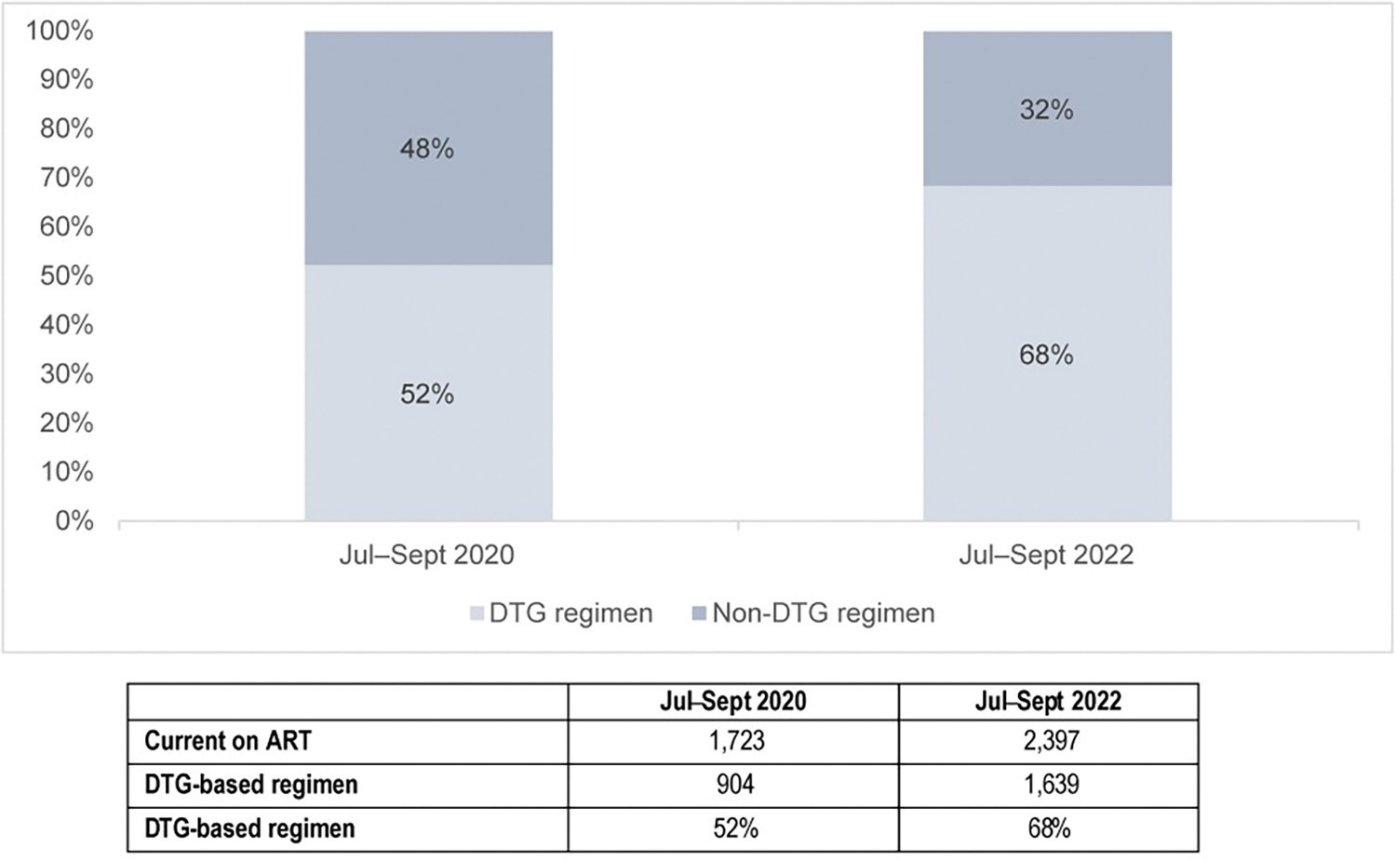
DTG regimen coverage among CLHIV ages 0–14 years, by quarter.

Following the rollout of specific interventions to scale up DTG coverage, the project reported a progressive increase in the percentage of CLHIV on a DTG-containing regimen, reaching 64% in July–September 2021 (data not shown) and 68% in July–September 2022 (Fig 1).

**Fig 2:**
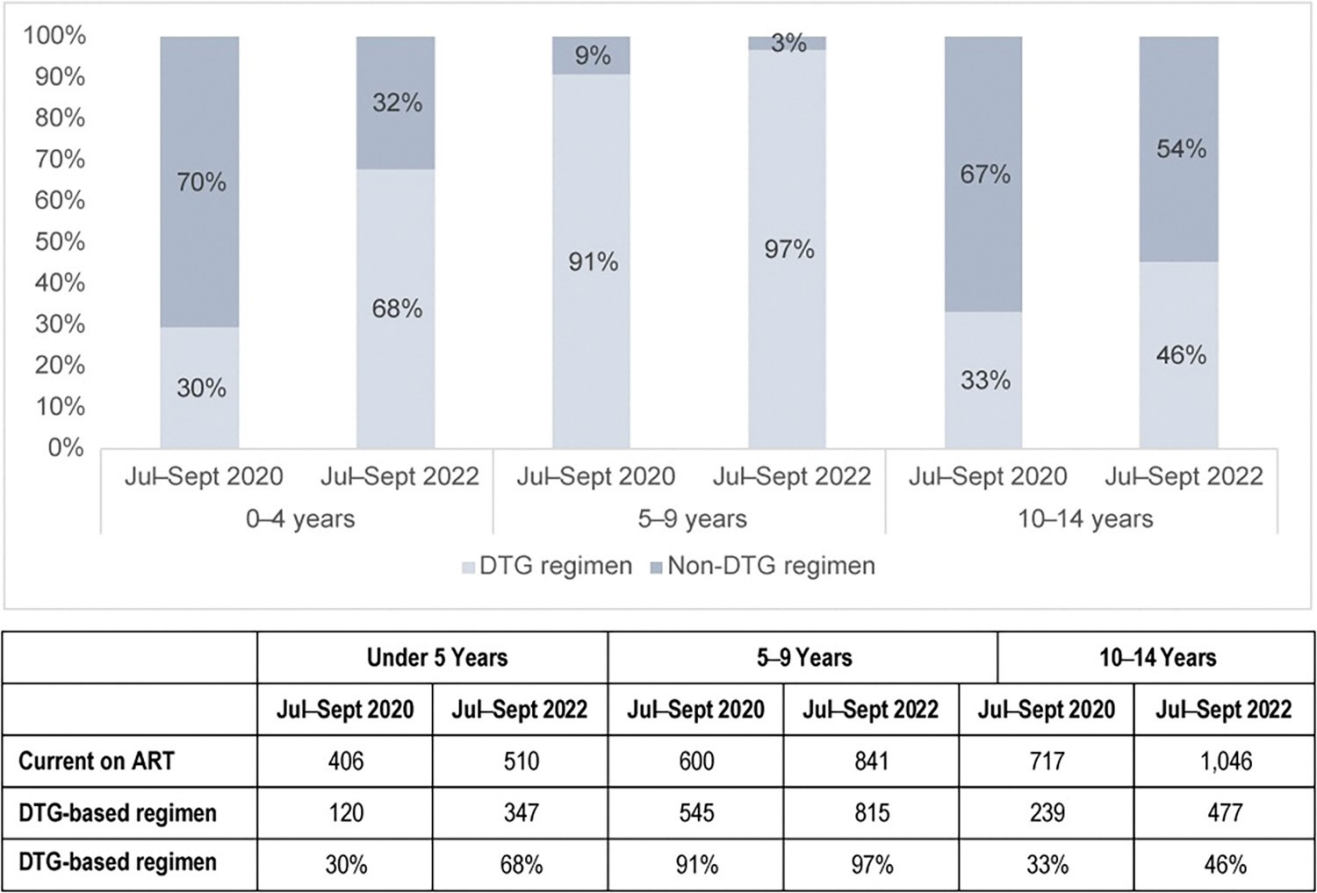
DTG regimen coverage among CLHIV, by age group and quarter.

When disaggregated by age group, about a third of the CLHIV ages under 5 years and ≥10 years were on a DTG-containing regimen (120 and 239 CLHIV, respectively), while those ages 5–9 years were already above 90% (545) at baseline. At endline, a three-fold increase in DTG coverage was observed among the youngest age group (under 5 years) with 347 CLHIV on DTG, the oldest age group (10–14 years) increased by one and a half (477 CLHIV on DTG), and coverage among those ages 5–9 years was approaching 100% (815 CLHIV on DTG) (Fig 2).

**Fig 3:**
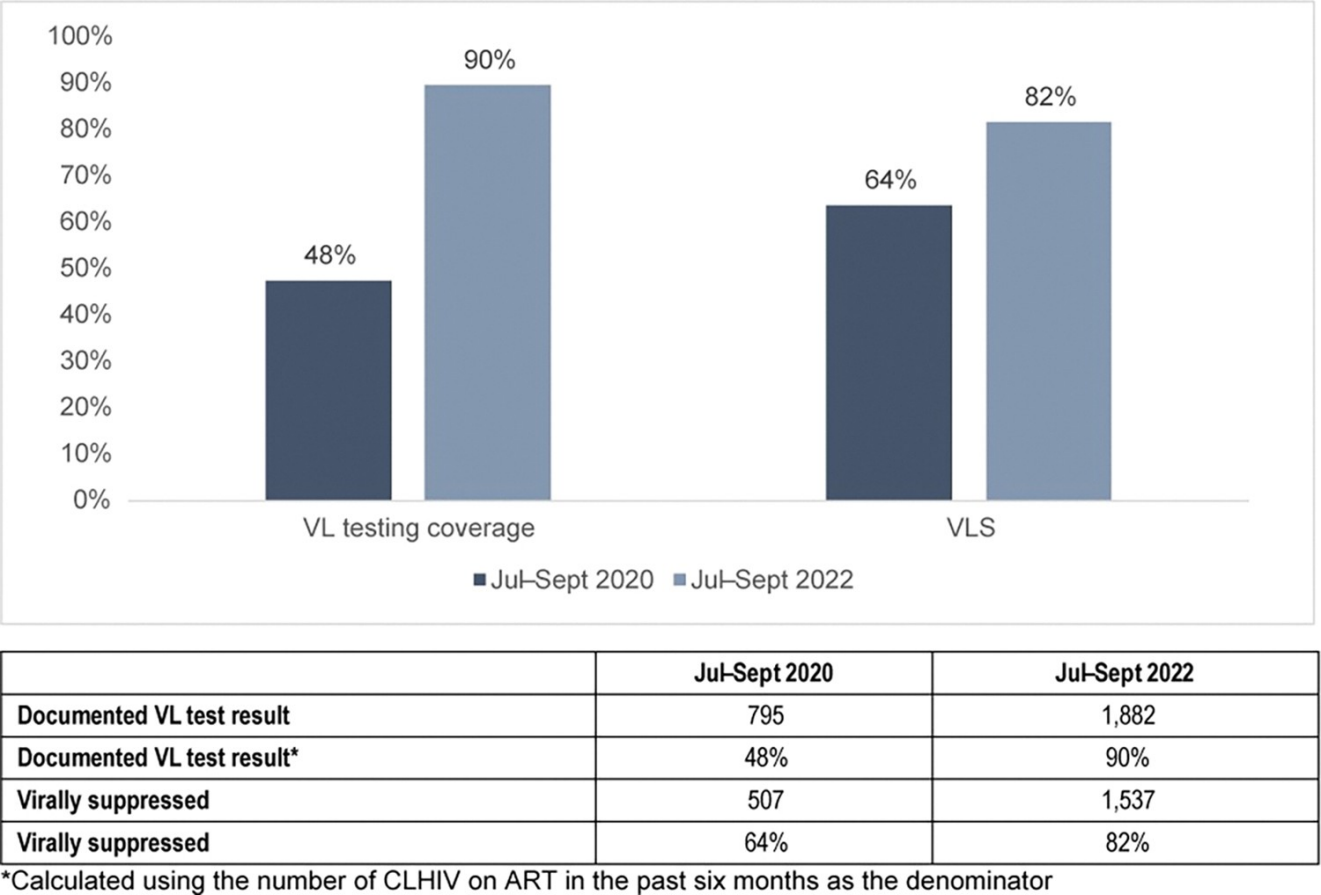
VL testing coverage and VLS among CLHIV, by quarter.

For VL testing coverage, at baseline 48% (795) of the 1,673 CLHIV ages 0–14 years who had been on treatment for at least six months had a documented VL test result within the past 12 months; at endline, VL testing coverage was 90% (1,882) among the 2,098 CLHIV ages 0–14 years who had been on treatment for at least six months. VLS also increased in this age group, from 64% (507) at baseline to 82% (1,537) at endline (Fig 3).

**Fig 4:**
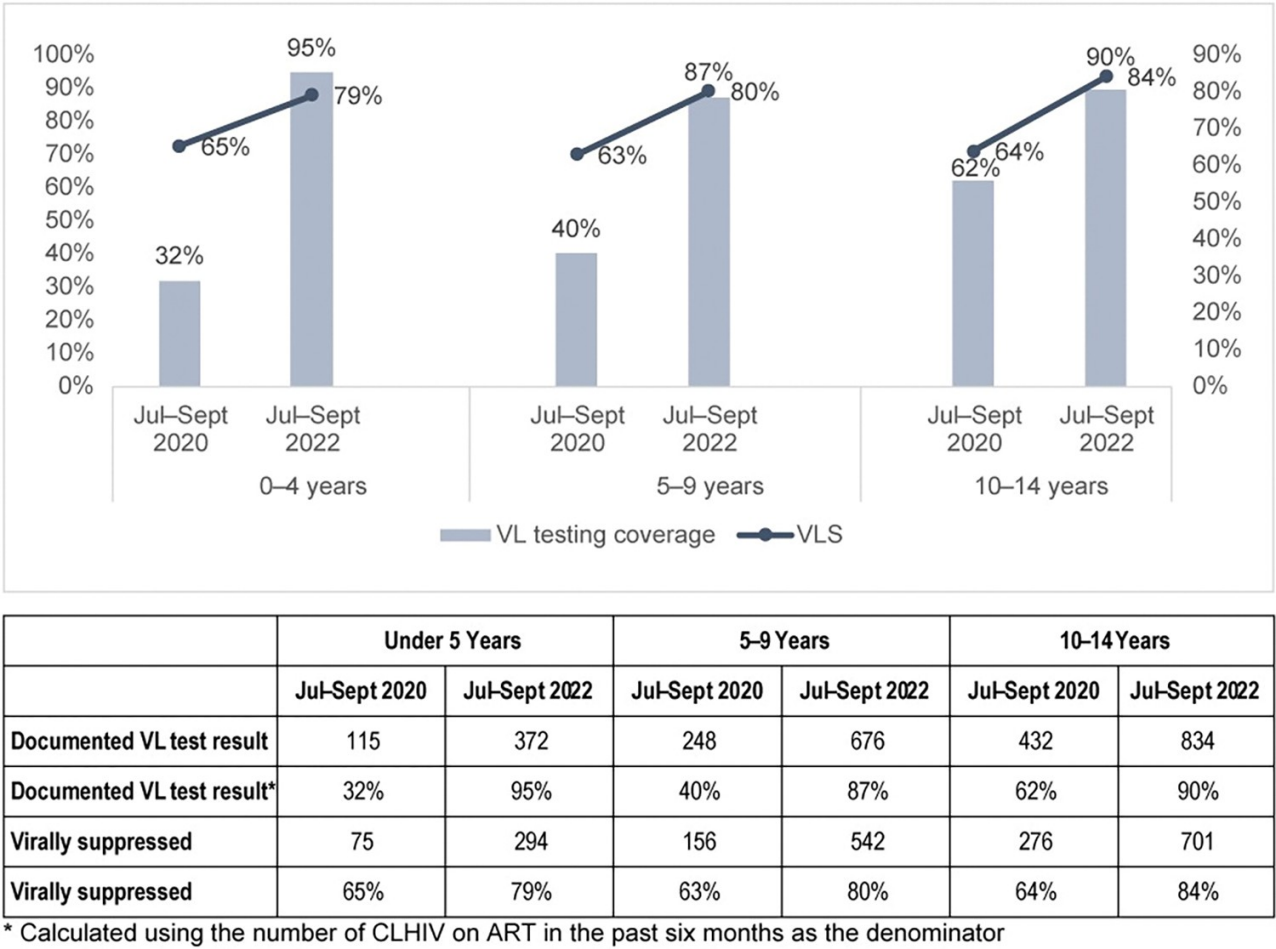
VL testing coverage and VLS among CLHIV, by age group and quarter.

When disaggregated by age group, only the youngest age group reached the 95% benchmark, while ages ≥5 years reached or approached 90% for VL testing coverage, with the highest proportional increase among those under 5 years. The proportional increase in VLS was similar across all three age groups, which also reported similar baseline VLS rates, and none of the age groups met the 95% benchmark for VLS at endline (Fig 4).

The proportion of CLHIV already on ART increased over time, from 58% at baseline to 71% at endline; similarly, MMD coverage increased from 57% at baseline to 85% at endline. When disaggregated by age group, new ART initiation was equally distributed across the three age groups, while the majority of follow-up clients were from the oldest age band. Among the CLHIV who were receiving MMD, about three-quarters were from the older age bands (5–9 years and 10–14 years) (Table 3).

**Table 3:** ART status and MMD coverage of CLHIV ages 0–14 years currently on treatment, at baseline and endline.

	Jul–Sept 2020 (baseline)		Jul–Sept 2022 (endline)	
**ART status**	**1,723**	**100%**	**2,397**	**100%**
**New ART initiations**	**714**	**42%**	**703**	**29%**
Under 5 years	219	31%	211	30%
5–9 years	228	32%	255	36%
10–14 years	267	37%	237	34%
**Follow-up ART clients**	**1,009**	**58%**	**1,694**	**71%**
Under 5 years	187	19%	299	18%
5–9 years	372	36%	586	34%
10–14 years	450	45%	809	48%
**MMD**	**1,723**	**100%**	**2,397**	**100%**
**Yes**	**976**	**57%**	**2,043**	**85%**
Under 5 years	176	18%	312	15%
5–9 years	299	31%	709	35%
10–14 years	501	51%	1,022	50%
**No**	**747**	**43%**	**354**	**15%**
Under 5 years	230	31%	198	56%
5–9 years	301	40%	132	37%
10–14 years	216	29%	24	6%

We also analyzed interruptions in treatment and death at baseline and endline. These remained stable over time at an average of 3.4% and 0.5%, respectively. No differences were observed when disaggregated by age group.

Data obtained from the national supply chain management system show a progressively increasing number of bottles of DTG, a decreasing volume of EFV 200 mg, and a relatively steady volume of EFV 600 mg over time (Table 4). These national-level data included the 29 sites of our analysis but also sites throughout the country.

**Table 4:** National-level data on number of bottles with a one-month supply of medication by ARV type, at baseline and endline.

	Jul–Sept 2020 (baseline)	Jul–Sept 2022 (endline)
DTG 10 mg	-	68,165
DTG 50 mg	24,093	113,157
EFV 200 mg	80,695	45,932
EFV 600 mg	5,730	34,683

All data, including those at the midline point of the analysis, are provided in S1 Dataset.

## Discussion

To our knowledge, this is the first analysis from West Africa that reports on interventions for scale-up of DTG and VL testing coverage, as well as to increase VLS among CLHIV ages 0–14 years. The data reported in this analysis show a progressive increase in DTG coverage among CLHIV from baseline to midline and endline. This increase coincides with the rollout of specific interventions as well as the availability of DTG 50 mg as of October 2020 and DTG 10 mg in March 2022, suggesting that the interventions may have played a role in the increase.

However, the overall increase in DTG coverage reported at endline was small (from 52% at baseline to 68% at endline), and the 80% benchmark was not achieved. This was possibly because the national guidelines did not permit DTG transition at the community level and CLHIV/caregivers were not actively contacted before their community-based ART refill appointments to request them to come to the ART clinic for DTG transition; instead, during their community refill the mediator and ART health care worker informed CLHIV/caregivers of the need to go to the health facility the following month for transition to DTG. If CLHIV/caregivers could not go to the health facility for their next ART refill appointment, the transition to DTG was further delayed. Addressing this structural barrier to DTG transition could help to increase DTG coverage in Togo.

Furthermore, since over time a higher proportion of CLHIV were already on treatment, the chance of not being transitioned to a DTG-containing regimen because of a missed appointment may have been higher than among treatment-naïve CLHIV, who were initiated on a DTG regimen at the time of their initial enrollment on ART services.

When disaggregated by age, at endline DTG coverage had more than doubled since baseline among CLHIV under 5 years. However, even with this two-fold increase, at endline this group had only reached 68% DTG coverage. This was likely the case because prior to March 2022, the lowest strength tablet of DTG available was 50 mg—five times the dose for CLHIV in this group—making providers reluctant to prescribe it. In March 2022, DTG 10 mg became available and was soon systematically prescribed by providers; however, our analysis ended in September 2022, before a significant increase could be observed.

The age group 5–9 years reached 97% DTG coverage at endline after already high coverage at baseline (91%). This age group was originally on a regimen containing ABC+3TC+EFV, but the country had a relatively small supply of EFV 200 mg in stock when DTG 50 mg became available, creating the opportunity to switch all CLHIV ages 5–9 years to a DTG-containing regimen.

The increase in DTG coverage was modest in the oldest age group, from 33% at baseline to 46% at endline. Due to a higher absolute number of CLHIV ages 10–14 years than those 5–9 years, the latter group reached higher proportional coverage faster. Moreover, although DTG has been shown to have better efficacy, tolerability, and durability than EFV, EFV has nonetheless been widely used as part of first-line treatment for HIV infection, including in Togo. At the time of the country’s transition to DTG, it still had a relatively large stock of EFV 600 mg, the strength suitable for CLHIV ages 10–14 years and the use of which slowed the DTG transition process.

Similarly, according to WHO, in 2021, several countries in Africa reported having large stocks of EFV that prevented or delayed the transition to DTG [9]. Strategies they adopted to manage the EFV stock and facilitate the transition to DTG included phasing out EFV procurement; using existing stock for patients stable on EFV or with contraindications to DTG; prioritizing DTG for new patients, pregnant and breastfeeding women, and patients with virological failure or toxicity on EFV; implementing MMD and differentiated service delivery models to reduce clinic visits and optimize drug supply; strengthening pharmacovigilance and monitoring systems to track drug utilization, adverse events, and resistance patterns; and engaging with stakeholders, including donors, manufacturers, civil society, and patients, to ensure alignment and coordination of the transition process.

The nonavailability of DTG in a strength lower than 50 mg until March 2022 emphasizes the critical role of supply chain management and forecasting systems and the importance of ability to access the correct pediatric formulations to support changes in ARV regimens as normative guidance evolves. In addition to these key enabling factors to ensure adequate stock of DTG for children, the availability and accessibility of DTG for children under 5 years has also been limited by factors such as low capacity and use of diagnostic tools to determine the weight and HIV status of children and a lack of skilled health workers and caregivers to administer and monitor DTG for children [9].

Quantifying the amounts of the formulations needed for CLHIV for procurement purposes requires data about the weight distribution of those children requiring ARV drugs, yet monthly stock monitoring in Togo relies upon age rather than weight, potentially leading to inaccurate forecasting. Hence, setting up a monitoring system that accurately documents this information or uses standard age-to-weight conversions to estimate the weight-band breakdown is a critical long-term goal [10].

Comparison of the overall increase in VL testing coverage, VLS, and DTG coverage showed a linear correlation and a proportionally similar increase of 1.8, 1.3, and 1.3 times, respectively. This VLS increase aligns with a large retrospective cohort study across six countries in Eastern and Southern Africa, indicating that DTG is effective in achieving VLS among CLHIV [11].

However, in our project, the 95% benchmark for VLS was not reached (82% at endline). One reason could be that barriers specific to in-person ART adherence counseling for CLHIV and caregivers and the lack of complementary virtual counseling offered limited how much adherence counseling CLHIV/caregivers received. Other factors included the low reported ART and VL literacy of caregivers, non-disclosure of HIV status to CLHIV by caregivers, and program inability to provide ART at the community level when CLHIV/caregivers could not come to the health facility.

When disaggregated by age, the VLS increase was similar across age groups while the increase in DTG coverage differed. Among CLHIV 5–9 years, the group where DTG coverage was highest and reached essentially all CLHIV currently on treatment, VLS may have been affected by low adherence to the medications. In the lower and upper age bands (under 5 years and 10–14 years), VLS may have been impacted by lower DTG coverage (68% and 46%, respectively). Before DTG 10 mg became available in March 2022, the challenges in using DTG 50 mg were also a likely factor for the younger age group.

The methods used to assess ART adherence—i.e., self-report and ARV refills—could have been misleading in health care worker decision-making around the frequency of adherence support provided, though similar methods have been used successfully in other studies conducted in Africa whereby adherence levels predicted through pharmacy refill were strongly associated with virological suppression [12,13].

ART adherence challenges for CLHIV/caregivers present the main barrier to achieving viral suppression among CLHIV, and delays in addressing them are likely to result in adverse treatment outcomes [14]. Studies have shown an increase in VLS with adherence counseling that assesses barriers to adherence and helps clients develop an adherence plan with concrete objectives that take into account psychological, emotional, and socioeconomic factors [15]. Given that CLHIV depend on caregivers in order to take the medication, it is critical to understand the nature of adherence behavior among caregivers and anticipate potential threats to optimal compliance. A qualitative study from Togo showed that lack of disclosure of positive HIV status and self-stigma negatively affected ART adherence among CLHIV [16]. The collection of qualitative data to learn caregivers’ experiences and perceptions around transition and adherence to the DTG-containing regimen is recommended for future research to identify barriers and develop solutions.

The provision of psychosocial support to caregivers after their children’s HIV diagnosis and during CLHIV’s first months on ART is crucial to prevent, identify, and address barriers to optimal treatment adherence [17,18]. In our project, over time the psychosocial support provided by the mediators shifted toward engaging expert patients and later mentor mothers, but the mediators themselves were not necessarily mothers of CLHIV, in contrast to other studies [19]. This may have negatively affected the support given to caregivers if the mediators did not have a full understanding of the challenges faced by mothers of CLHIV. In turn, this could have affected the children’s adherence and attainment of VLS.

Viral suppression can be much lower among children than adults, including because children rely on others to administer their medications and ensure clinic attendance, and caregivers often face psychosocial and economic barriers that directly affect child clinical outcomes. Fidelity to and reinforcement of adherence strategies, as well as efforts to improve ART and VL literacy and provide psychosocial support to caregivers, is key, particularly when the switch to DTG is accelerated.

No differences in interruption in treatment were observed over time, suggesting that increased DTG coverage did not affect it. Likewise, deaths were close to 0% across all time points and ages, making it unlikely that increased DTG coverage had any impact.

Of note, the “solution” interventions that contributed to the scale-up of DTG coverage also led to several spillover effects. First, as CLHIV were initiated on or transitioned to a DTG-containing regimen, they were also line listed for subsequent VL testing, helping to increase VL testing coverage. In addition, the enhanced ART adherence support provided contributed to increased rates of VLS. In turn, the higher number of virally suppressed CLHIV was correlated with more CLHIV eligible for MMD and resulted in higher MMD coverage.

MMD has been found a feasible model among CLHIV in sub-Saharan Africa [20]. Our analysis showed that at endline, PEPFAR’s 80% MMD coverage benchmark had been attained, creating a strong basis for CLHIV on a DTG-containing regimen to sustain viral suppression, as demonstrated in other studies [21]. However, since the VLS benchmark was not reached, it is possible that the CLHIV were on suboptimal doses due to failure to adjust dosing with increasing weight. This lends support to the WHO-recommended use of weight bands in DTG dosing for children as vital for MMD implementation in this population [8,22,23].

This analysis also highlights the key role of routine patient-level data use to successfully monitor transitions to new ARVs. This represents important information for the national HIV response in Togo, as other countries such as Kenya have already enhanced the national health information management system to determine eligible clients and prioritize the switch [24].

Further research is needed in non-trial settings to identify barriers to DTG transition among CLHIV under 5 years and 10–14 years.

### Limitations

The main limitations of this analysis were the use of aggregated data such that we could not account for CLHIV who were already virally suppressed at the time they were transitioned to a DTG-containing regimen. In addition, caregiver characteristics were not known, self-/caregiver report was used to assess CLHIV adherence to treatment, and participation in the individual/group ART adherence counseling, which may have had an impact on VLS, was not routinely documented. In addition, the significant amount of missing CD4 data in our study did not allow for accurate determination of the proportion of CLHIV ≥5 years with advanced HIV disease, which may have resulted in poor treatment outcomes.

We also acknowledge as a limitation that our use of aggregated data and our inability to account for confounding factors precluded the option to conduct a statistical analysis to test the causal relationship between the intervention and DTG coverage. We recommend that future studies address this area.

An understanding of CLHIV and caregiver experiences of the switch to DTG-containing regimens is critical to inform programming and policy decisions intended to promote uptake and adherence to a new treatment, though that information was not included in our analysis. Other studies conducted among adult patients found that when switched to DTG during a routine visit in which they had expected their regular prescription they felt rushed, and some felt unprepared for the abrupt change in treatment schedule and to handle new adverse events associated with DTG [25].

Although site-level stock of DTG was regularly monitored, we could only access national-level data for stock of DTG and EFV. As a result, we were unable to substantiate site-level feedback suggesting that large stock of EFV was a bottleneck to DTG scale-up. We also could not assess whether the shortages of DTG at the site level negatively affected the scale-up of DTG-containing regimens.

## Conclusions

Our findings suggest that in this setting, implementation of a standardized package of interventions designed to respond to specific bottlenecks is feasible and can successfully scale up DTG-containing regimens as part of ART optimization strategies for CLHIV. Furthermore, the solutions employed to scale up DTG resulted in other important spillover effects, such as increased VL testing coverage, VLS, and MMD coverage.

We believe that our multifaceted, combined approaches, which included provision of comprehensive multidisciplinary care (e.g., mediator, nurses, clinicians, pharmacists, laboratory technician, data manager, and project coordinator), the mediator-led educational and supportive strategies focused on CLHIV and caregiver needs, VL monitoring strategies, regimen-related strategies, differentiated service delivery strategies (e.g., MMD), and data-driven decision-making are more effective than one specific intervention. Additionally, the solutions which focused on optimizing the use of social and community support services represent an important aspect for sustainability of the interventions and long-term epidemic control.

However, the overall moderate increase in DTG coverage specifically among children under 5 years and ≥10 years emphasizes the importance of a robust supply chain management system, ARV stock visibility and documentation at the site level, and the need to capacitate and empower the health care workers to prescribe DTG to all eligible CLHIV as one of the most effective and safe ART options currently available.

Studies that use individual-level data and data on advanced HIV disease, VL status at DTG initiation, caregiver characteristics, adherence, counseling services, MMD, and DTG supply availability, will be critical to better understand the factors that can drive scale-up of DTG coverage among CLHIV.

## Supporting information

S1 Dataset #EAWA Togo data and graphs for July–September 2020 (baseline), July–September 2021 (midline), and July–September 2022 (endline). (XLSX)
